# RARRES1 attenuates H_2_O_2_-induced RPE cell injury and inhibits choroidal neovascularization

**DOI:** 10.3389/fphys.2025.1641653

**Published:** 2025-10-16

**Authors:** Yimeng Li, Caixia Wang, Tao Deng, Xuejing Li, Renhao Xu, Qingli Shang

**Affiliations:** ^1^ Department of Ophthalmology, The Second Hospital of Hebei Medical University, Shijiazhuang, Hebei, China; ^2^ Department of Anesthesiology, The Second Hospital of Hebei Medical University, Shijiazhuang, Hebei, China; ^3^ Department of Neurology, The Second Hospital of Hebei Medical University, Shijiazhuang, Hebei, China

**Keywords:** neovascular age-related macular degeneration, RARRES1, choroidal neovascularization, viability, oxidative stress

## Abstract

**Introduction:**

Neovascular age-related macular degeneration (nAMD) is a leading cause of vision loss in the elderly, yet its underlying molecular mechanisms remain incompletely understood, and novel biomarkers and therapeutic targets are urgently needed. This study aimed to identify and functionally characterize potential biomarkers and therapeutic candidates for nAMD, with a focus on retinoic acid receptor responder protein 1 (RARRES1).

**Methods:**

Tandem mass tag (TMT)–based proteomic analysis was performed on aqueous humor samples from patients with nAMD and age-related cataracts. RARRES1 expression was examined in aqueous humor, laser-induced choroidal neovascularization (CNV) model mice, and human ARPE-19 cells exposed to H_2_O_2_. Functional studies assessed the effects of RARRES1 on oxidative stress, cell death, inflammatory and angiogenic factor expression, and signaling pathways in ARPE-19 cells. Its effects on proliferation, migration, and tube formation were tested in HUVECs. *In vivo*, a RARRES1-overexpressing AAV2 vector was injected intraocularly into CNV model mice, and lesion size and vascular leakage were evaluated using fundus fluorescein angiography, hematoxylin and eosin staining, and isolectin B-4 fluorescence staining.

**Results:**

RARRES1 was significantly reduced in the aqueous humor of nAMD patients, in CNV model mice, and in H_2_O_2_-treated ARPE-19 cells. Overexpression of RARRES1 in ARPE-19 cells mitigated oxidative stress–induced damage, suppressed inflammatory and angiogenic factor expression, inhibited JNK phosphorylation, and increased Sirtuin 1 and Nrf2 expression. In HUVECs, RARRES1 reduced proliferation, migration, and tube formation. *In vivo*, intraocular delivery of RARRES1 significantly reduced CNV lesion size and vascular leakage.

**Conclusion:**

RARRES1 protects retinal pigment epithelial cells from oxidative stress, inhibits choroidal neovascularization, and modulates inflammatory and angiogenic pathways. These findings identify RARRES1 as a potential biomarker and therapeutic target for nAMD.

## Introduction

Age-related macular degeneration (AMD) ranks among the primary causes of vision loss in older adults, with an estimated 288 million individuals expected to be affected globally by 2040 (Wong et al., 2014). Neovascular AMD (nAMD), distinguished by choroidal neovascularization (CNV), affects around 10% of individuals with AMD but contributes to 90% of the disease’s most severe vision loss cases (Dimopoulos et al., 2013). Although the underlying causes of nAMD are not yet completely defined, it is known to involve complex and multifactorial processes, including aging, oxidative stress, angiogenesis, inflammation, and dysregulated autophagy.

For many patients, anti-vascular endothelial growth factor (VEGF) therapy has brought significant gains in visual function; however, several limitations remain, such as incomplete or suboptimal responses, disease recurrence, and the need for frequent intravitreal injections (Chakravarthy and Peto, 2020). Thus, additional studies are imperative to clarify the molecular basis of nAMD progression and to facilitate the development of new therapies.

The expression of retinoic acid receptor responder 1 (RARRES1) can be upregulated by tazarotene and retinoic acid receptors. RARRES1 shows extensive expression in numerous tissues and organs, including the gastrointestinal tract, liver, kidney, prostate, endometrium, head and neck, and nasopharynx, where it regulates cellular functions (Jing et al., 2002; Kwok et al., 2009; Son et al., 2009; Tokumaru et al., 2005; Youssef et al., 2004). RARRES1 serves as a tumor suppressor gene, with its expression often diminished in various malignancies, where it influences cell migration, growth, apoptosis, and the prevention of angiogenesis (Guo et al., 2024; Huebner et al., 2017; Ma et al., 2022; Roy et al., 2017; Wang et al., 2021). Moreover, RARRES1 is involved in multiple biological processes—such as inflammation, oxidative stress, fibrosis, fatty acid metabolism, and autophagy—that are also critically associated with the pathogenesis of nAMD. Despite these connections, research on RARRES1 in ophthalmic diseases remains limited, and, to date, no studies have explored its role in nAMD.

Herein, this study aims to explore the role of RARRES1 in nAMD.

## Materials and methods

### Participants

nAMD patients (n = 8) and age-related cataracts as controls (n = 8) were recruited from the Ophthalmology Department of the Second Hospital of Hebei Medical University. Informed written consent was obtained from each participant. The study was approved by the Ethics Committee of the Second Hospital of Hebei Medical University. The detailed clinical characteristics of the participants were shown in Supplementary Table S1. No significant differences between the two groups in terms of age, sex, BMI, smoking, or alcohol consumption were observed.

The inclusion criteria were as follows: (1) age over 60 years; (2) a first-time diagnosis of nAMD based on the Age-Related Macular Degeneration Preferred Practice Pattern (Flaxel et al., 2020), or a diagnosis of cataract requiring surgical treatment; and (3) no history of ocular treatments such as intravitreal anti-VEGF injections, photodynamic therapy, or photocoagulation.

Exclusion criteria were as follows: (1) the existence of ocular diseases other than cataracts or nAMD; (2) a history of intraocular disease or prior ocular surgery; and (3) the presence of serious systemic conditions such as cancer, autoimmune diseases, congestive heart failure, hypertension, or diabetes mellitus.

Aqueous humor samples were collected from nAMD patients prior to their first intravitreal injection of anti-VEGF drugs, and from control subjects during cataract surgery. Using a 1 cc syringe and a cannula, 50–150 μL of aqueous humor was obtained from each participant and immediately transferred into Eppendorf tubes, then stored at −80 °C. For proteomic sequencing, every four individual samples in each group were randomly pooled and mixed, resulting in two paired samples.

### Quantitative proteomic analysis via tandem mass tag (TMT) technology

Shanghai Applied Protein Technology Co., Ltd. conducted the TMT-based quantitative proteomic analysis, following the procedures detailed in previous research (Liu et al., 2021). Mascot 2.2 and Proteome Discoverer 1.4 were utilized for protein identification and quantification.

### Cell culture and transfection

Human umbilical vein endothelial cells (HUVECs) were cultured in endothelial cell medium, supplemented with 10% fetal bovine serum (FBS, Gibco) and ECGS (ECM, ScienCell), at 37 °C in a humidified 5% CO_2_ atmosphere. ARPE-19 cells (ATCC) were grown in DMEM/F12 medium containing 10% FBS.

Transfection was performed once the cells attained 70%–80% confluence. RARRES1 small interfering RNA (si-RARRES1) and si-NC (negative control siRNA), as well as pcDNA3.1 control vector and the pcDNA3.1-RARRES1 overexpression plasmid, were designed and provided by GenePharma. Lipofectamine 2000 (Invitrogen) was employed for transfection, following the manufacturer’s instructions. The siRNA or plasmid complexes were premixed and added to cells in 6-well plates. The final concentration of siRNA was 50 nM, and plasmids were transfected at a concentration of 2 µg per well. The medium was exchanged for fresh culture medium with 10% FBS and no antibiotics, 6 h post-transfection.

HUVECs were collected for subsequent assays 48 h after transfection. When ARPE-19 cells reached 90%–95% confluence, they were starved in medium containing 1% FBS for 24 h, followed by H_2_O_2_ treatment for 48 h. H_2_O_2_ was freshly prepared at a concentration of 200 µM and added directly to the culture medium. Samples were then collected for subsequent analysis.

### Cell viability assay

Cell viability was measured with the Cell Counting Kit-8 (CCK-8; Beyotime). Briefly, cells were seeded in 96-well plates at a density of 5 × 10^3^ cells per well in 100 μL of complete medium. After 48 h of transfection, 10 μL of CCK-8 solution was added to each well. The cells were then incubated at 37 °C for 1 h. Cell viability was determined by measuring the optical density at 450 nm using a microplate reader (BioTek). Blank wells containing medium and CCK-8 without cells were used to subtract background absorbance.

### Transwell migration assays

For the Transwell assay, serum-starved transfected HUVECs (5 × 10^4^ cells/well) were placed in the upper chamber (8 μm pore size, 24-well plate; Corning), with the lower chamber filled with medium containing 1% FBS. Cells were suspended in 200 μL of serum-free medium before seeding into the upper chamber. After 24 h of incubation at 37 °C in a humidified atmosphere with 5% CO_2_, the cells that had migrated to the lower surface of the membrane were fixed with 4% paraformaldehyde for 15 min and stained with 0.1% crystal violet for 20 min. Non-migrated cells on the upper surface were gently removed using a cotton swab. the number of migrated cells were visualized and counted under a light microscope (Olympus).

### Tube formation assay

Matrigel (Cat# 356230, Corning) was thawed overnight on ice, aliquoted into precooled 96-well plates (60 μL per well), and incubated at 37 °C for 30 min. HUVECs were transfected with siRNA or plasmid cDNA for 48 h, after which they were seeded on Matrigel at a density of 5 × 10^3^ cells per well in 100 μL of complete endothelial basal growth medium (EBM, supplemented with EGM SingleQuots, Lonza), and the cells were cultured at 37 °C in a 5% CO_2_ atmosphere. After 18 h of incubation, tube-like structures were observed, and phase-contrast images were captured using a microscope (Olympus). Tube formation was evaluated with ImageJ software.

### Annexin V-FITC/PI staining

Apoptosis in ARPE-19 cells was assessed using an Annexin V-FITC Apoptosis Detection Kit (Cat#: E-CK-A217, Elabscience, Wuhan, China), following the manufacturer’s protocol. After reaching approximately 75% confluence, cells were exposed to 400 μmol/L H_2_O_2_ for 48 h to induce oxidative stress. Following treatment, cells were gently washed twice with cold PBS and harvested using trypsinization without EDTA to preserve membrane integrity. Cells were then resuspended in 1× binding buffer at a concentration of 1 × 10^6^ cells/mL. A total of 100 µL of the cell suspension was incubated with 5 µL of Annexin V-FITC and 5 µL of propidium iodide (PI) in the dark at room temperature for 15 min. Apoptotic cells were analyzed using a FACS LSRFortessa flow cytometer (BD Biosciences) within 1 h of staining.

### Measurement of reactive oxygen species (ROS) levels

Intracellular ROS levels were measured using DCFH-DA dye (Cat#: E-BC-K138-F, Elabscience). Cells were incubated with DMEM containing 10 µM DCFH-DA for 30 min at 37 °C in the dark to prevent photobleaching. After incubation, cells were washed three times with serum-free DMEM to remove excess dye. The labeled cells were subsequently viewed using a fluorescence microscope (Nikon) using an excitation wavelength of 488 nm and an emission wavelength of 525 nm.

### Animal care and laser-induced CNV model

C57BL/6 male mice (6–8 weeks old) (Beijing HFK Bioscience) were housed under SPF conditions. They were provided with ad libitum access to food and water and maintained on a 12-h light/dark cycle. All animal experiments were approved by the Ethics Committee of the Second Hospital of Hebei Medical University.

Laser-induced CNV was induced in the mice following previously described protocols (Wang et al., 2020). The mice were anesthetized with chloral hydrate, and 0.5% tropicamide (Henry Schein) was applied to dilate the pupils. Laser photocoagulation was performed using an ophthalmic laser system (Nd, 532 nm; Coherent Novus Omni) with a 50 μm spot diameter, 100 ms duration, and 300 mW power. In each eye, five laser spots were applied around the optic disc, and the successful disruption of Bruch’s membrane was confirmed by the formation of a bubble.

### Intraocular injections of adeno-associated virus vectors

Using a Harvard Apparatus microinjector and pulled glass micropipettes, intravitreal injections were performed as outlined in the previous description (Mori et al., 2001). Using a dissecting microscope, the sharpened tip of the micropipette was inserted through the sclera just behind the limbus into the vitreous cavity. Each micropipette was calibrated to deliver 2 μL of the vehicle, which contained around 1 × 10^13^ viral genome copies (vgc) of AAV2-Rarres1 and AAV2-ZsGreen, when the foot switch was activated.

### Hematoxylin and eosin (HE) staining and choroidal flat mounting

The eyeballs were enucleated 7 days after laser injury and fixed in eyeball fixative solution for 24 h at 4 °C. After dehydration through graded ethanol (70%, 80%, 95%, and 100%) and embedding in paraffin, the eye tissues were sectioned to a thickness of 4 μm using a microtome. The sections were stained with hematoxylin and eosin (HE) following standard protocols and mounted with neutral gum for histological evaluation under a light microscope.

Seven days post-photocoagulation, the CNV lesion size was quantified using immunofluorescence staining of choroidal flat mounts with IB4 (FITC-IB4; Sigma-Aldrich). The flat mounts were incubated to specifically label endothelial cells within CNV lesions. After staining, samples were washed and mounted with antifade mounting medium.

### Quantitative PCR (qPCR)

TRIzol reagent (Takara) was used to extract total RNA from treated ARPE-19 cells or retinal tissues. The purity and concentration of RNA were assessed by measuring absorbance at 260/280 nm using a NanoDrop spectrophotometer (Thermo Fisher Scientific). RNA integrity was confirmed by agarose gel electrophoresis. cDNA synthesis was performed with the PrimeScript 1st strand cDNA synthesis kit (Monad Biotech). mRNA levels were quantified with a SYBR qRT-PCR kit (Monad Biotech) on a CFX Connect Real-Time PCR System (Bio-Rad). Gene expression was determined using the 2^−ΔΔCT^ method, normalizing to GAPDH mRNA. Primer sequences for qRT-PCR are provided in Supplementary Table S2.

### Western blotting

The cells or triturated RPE, choroid, or scleral tissues were lysed in RIPA buffer on ice and then boiled in SDS loading buffer. The resulting proteins were resolved by 6%–12% SDS-PAGE and transferred onto nitrocellulose membranes. The membranes were blocked with 5% skim milk, cut horizontally when needed, and incubated overnight at 4 °C with the selected primary antibodies. The primary antibodies include RARRES1 (sc-390461, Santa Cruz), phospho-JNK (HY-P80831, MCE), JNK (HY-P80197, MCE), phospho-ERK (HY-P81156, MCE), ERK (HY-P80393, MCE), Nrf-2 (80593-1-RR, Proteintech), SIRT-1 (HY-P80319, MCE), Occludin (502601, ZEN), β-actin (HRP-66009, Proteintech). The membranes were washed and then incubated with the corresponding secondary antibodies for 1 h at room temperature. Protein bands were visualized with an enhanced chemiluminescence (ECL) reagent and analyzed using a Bio-Rad GelDoc imaging system. If necessary, the membranes were stripped with stripping buffer (Solarbio) for 30 min at room temperature, and additional proteins were detected on the same membranes.

### Fundus fluorescein angiography (FFA)

Seven days after laser photocoagulation, CNV model mice were anesthetized with an intraperitoneal injection of a ketamine (80 mg/kg) and xylazine (10 mg/kg) mixture. Pupils were dilated using topical application of 1% tropicamide and 2.5% phenylephrine eye drops. Fluorescein sodium (Cat# HJ20130649; Lisheng Pharmaceutical) was intraperitoneally injected at a dose of 5 μg/g body weight. Fluorescein angiography (FFA) images were captured using a retinal angiography system (Heidelberg Engineering).

### Statistics

Using Prism 9 (GraphPad Software) and SPSS software, statistical analyses were carried out. Data are expressed as means ± standard deviations (SD) from at least three independent experiments. Brown-Forsythe ANOVA with Dunnett’s T3 test was used to compare the multiple groups, and t-test was used to compare two groups. A p-value of less than 0.05 was deemed statistically significant.

## Results

### Proteomic profiling of aqueous humor reveals RARRES1 downregulation in nAMD patients

**FIGURE 1:**
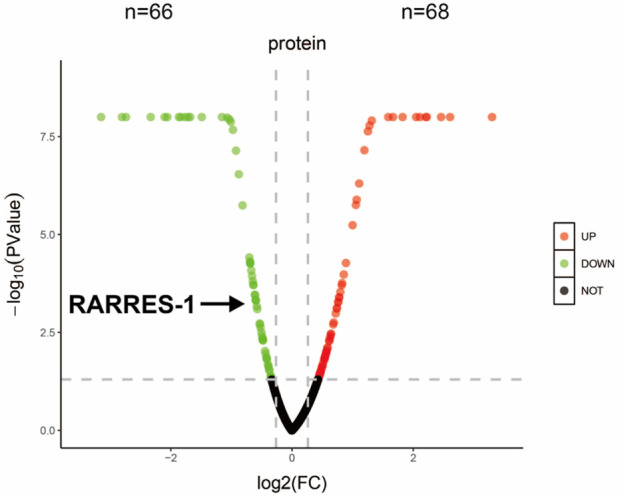
Proteomic profiling and bioinformatic analysis. Volcano plot of all identified proteins. Red and green dots represent significantly upregulated and downregulated proteins in the nAMD group compared to the cataract group (Student’s t-test, P < 0.05, |log_2_ fold change| > 1.2). RARRES1 was identified as a moderately downregulated candidate biomarker.

Proteomic analysis of aqueous humor samples from nAMD patients identified 1,241 expressed proteins, with 134 of them being differentially expressed-68 upregulated and 66 downregulated-based on a threshold of P < 0.05 and a log2 fold change >1.2 or <0.83 (Student’s t-test). Notably, RARRES1 was one of the significantly downregulated proteins in the aqueous humor of nAMD patients (Figure 1). This finding suggests that RARRES1 may play a critical role in the pathophysiology of nAMD, warranting further functional investigation.

### RARRES1 expression is downregulates in H_2_O_2_ -treated ARPE-19 cells

**FIGURE 2:**
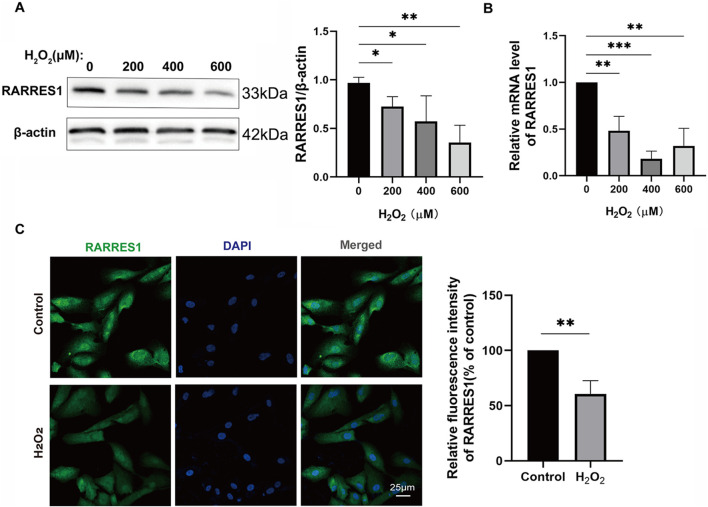
RARRES1 Expression in ARPE-19 Cells. **(A,B)** Western blot and qPCR analyses of RARRES1 protein and mRNA levels in ARPE-19 cells treated with various concentrations of H_2_O_2_. Both assays showed a significant decrease in RARRES1 expression in H_2_O_2_-treated cells compared to control groups. **(C)** Immunofluorescence analysis of RARRES1 expression in H_2_O_2_-treated ARPE-19 cells confirmed the reduced expression observed in biochemical assays. Protein levels were normalized to β-actin, and mRNA levels were normalized to GAPDH. Data are presented as mean ± SD; n = 3. *P < 0.05, **P < 0.01, ***P < 0.001.

To explore the expression profile of RARRES1 in ARPE-19 cells under both normal and oxidative stress conditions, we conducted comparative analyses using Western blotting, qPCR, and immunofluorescence staining. ARPE-19 cells were treated with different concentrations of H_2_O_2_ (0, 200, 400, and 600 μM) for 48 h for both Western blot and qPCR analyses. Western blot results revealed a significant downregulation of RARRES1 protein expression in H_2_O_2_-treated ARPE-19 cells compared to the control group (0 μM) (Figure 2A). This was further confirmed by qRT-PCR, which showed a marked reduction in RARRES1 mRNA levels following H_2_O_2_ exposure (Figure 2B). Immunofluorescence analysis of cells treated with 400 μM H_2_O_2_ revealed significantly weaker RARRES1 fluorescence intensity compared to untreated controls (Figure 2C). Notably, this expression pattern aligns with our previous findings of reduced RARRES1 levels in aqueous humor samples from patients with nAMD. These results demonstrate that oxidative stress directly suppresses RARRES1 expression in retinal pigment epithelial cells, paralleling clinical observations in nAMD.

### RARRES1 attenuates H_2_O_2_-induced oxidative stress and protects ARPE-19 cells

**FIGURE 3:**
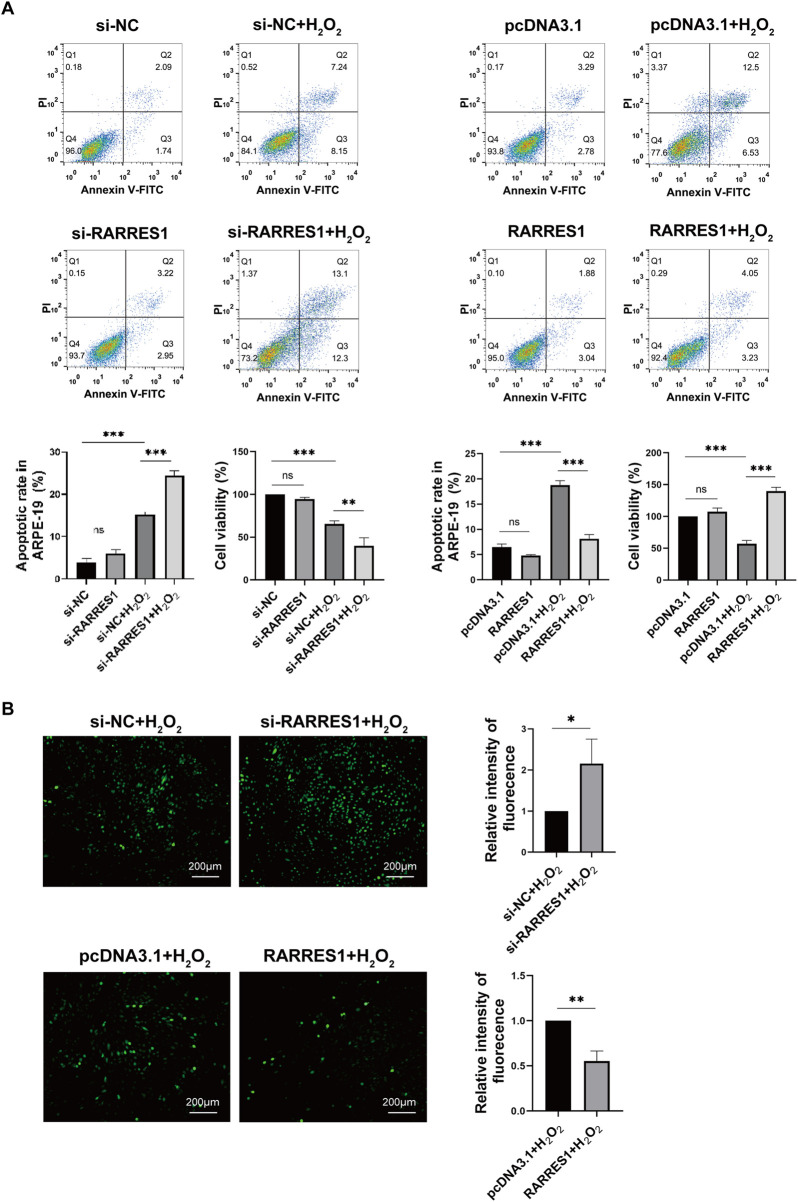
The Role of RARRES1 in H_2_O_2_-Treated ARPE-19 Cells. **(A)** Apoptosis and cell viability were assessed using flow cytometry and the CCK-8 assay across four groups: RARRES1 knockdown (si-RARRES1), siRNA negative control (si-NC), RARRES1 overexpression (RARRES1), and vector control (pcDNA3.1). All groups were treated with 400 μM H_2_O_2_(+H_2_O_2_). Overexpression of RARRES1 significantly reduced apoptosis and increased cell viability in H_2_O_2_-treated ARPE-19 cells. **(B)** Intracellular ROS production was decreased in H_2_O_2_-induced ARPE-19 cells with RARRES1 overexpression, while RARRES1 knockdown had the opposite effect. Scale bar = 100 μm. Data are presented as mean ± SD; n = 3. *P < 0.05, **P < 0.01, ***P < 0.001, ns = not significant.

To determine whether RARRES1 regulates H_2_O_2_-induced oxidative stress in ARPE-19 cells, we knocked down RARRES1 using siRNA and overexpressed RARRES1 using the pcDNA3.1 plasmid. Cells transfected with negative control siRNA (si-NC) and pcDNA3.1 served as the respective controls. RARRES1 expression was notably enhanced in comparison to the negative control group (Supplementary Figure S1). To assess whether RARRES1 protects ARPE-19 cells from H_2_O_2_-induced damage, we performed flow cytometry to analyze apoptosis and conducted CCK-8 assays. The results showed that knockdown of RARRES1 significantly increased H_2_O_2_-induced apoptosis, with fewer cells surviving H_2_O_2_ exposure in the RARRES1 knockdown group compared to the si-NC group. Conversely, overexpression of RARRES1 significantly suppressed H_2_O_2_-induced apoptosis and improved cell viability compared to the pcDNA3.1 group (Figure 3A). The DCFH-DA fluorescent probe was used to measure ROS production. As shown in Figure 3B, knockdown of RARRES1 increased ROS generation in ARPE-19 cells, whereas overexpression of RARRES1 reduced ROS production. These data indicate that RARRES1 plays a protective role against oxidative stress by reducing ROS levels and apoptosis in RPE cells.

### RARRES1 modulates oxidative stress-induced inflammation, angiogenesis, and RPE barrier integrity in ARPE-19 cells

**FIGURE 4:**
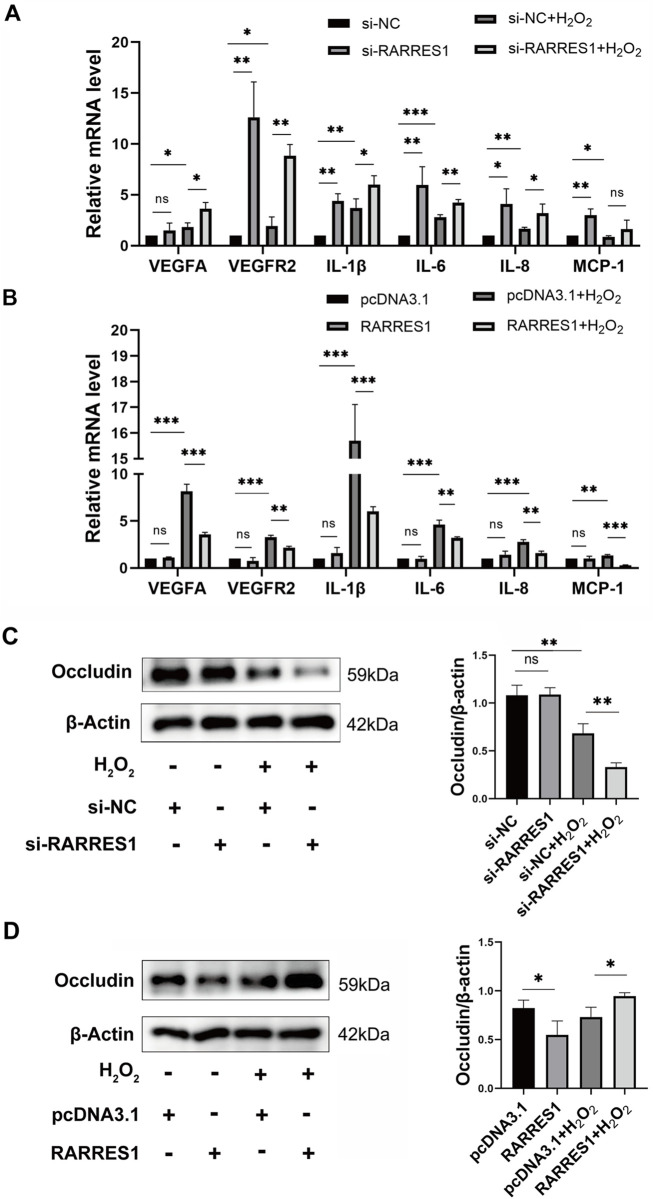
Effects of RARRES1 on Inflammatory and Angiogenic Factor Expression in ARPE-19 Cells. **(A)** Knockdown of RARRES1 significantly increased the mRNA expression of pro-angiogenic and pro-inflammatory factors, including VEGFA, VEGFR2, IL-1β, IL-6, IL-8, and MCP-1, compared to the scramble siRNA control group. **(B)** Western blot analysis showed a reduction in Occludin protein levels in ARPE-19 cells following RARRES1 knockdown, compared to the pcDNA3.1 control group. Conversely, RARRES1 overexpression restored Occludin expression. Protein levels were normalized to β-actin, and mRNA levels were normalized to GAPDH. Data are presented as mean ± SD. *P < 0.05, **P < 0.01, ***P < 0.001, ns = not significant.

To investigate whether RARRES1 is involved in inflammation and angiogenesis, we used qPCR to examine the expression of proangiogenic and inflammatory factors. It was found that knockdown of RARRES1 significantly upregulated the mRNA expression of VEGFA, VEGFR2, IL-1β, MCP-1, IL-8, and IL-6 in the presence of H_2_O_2_ (Figure 4A). In contrast, overexpression of RARRES1 significantly downregulated the mRNA levels of these genes in H_2_O_2_-insulted cells (Figure 4B).

Next, we assessed the protein expression of the tight junction protein Occludin via Western blot analysis. Notably, RARRES1 knockdown significantly reduced Occludin expression (Figure 4C), whereas RARRES1 overexpression restored Occludin levels in H_2_O_2_-insulted cells (Figure 4D).

The conditioned medium from RARRES1 knockdown cells further enhanced the angiogenic potential of HUVECs, as evidenced by increased cell numbers and junction formation (Supplementary Figure S2).

Together, these findings suggest that RARRES1 not only suppresses inflammation and angiogenic signaling but also helps maintain RPE barrier integrity under oxidative stress conditions.

### RARRES1 overexpression suppresses JNK phosphorylation while upregulating SIRT1 and Nrf2 in H_2_O_2_-treated ARPE-19 cells

**FIGURE 5:**
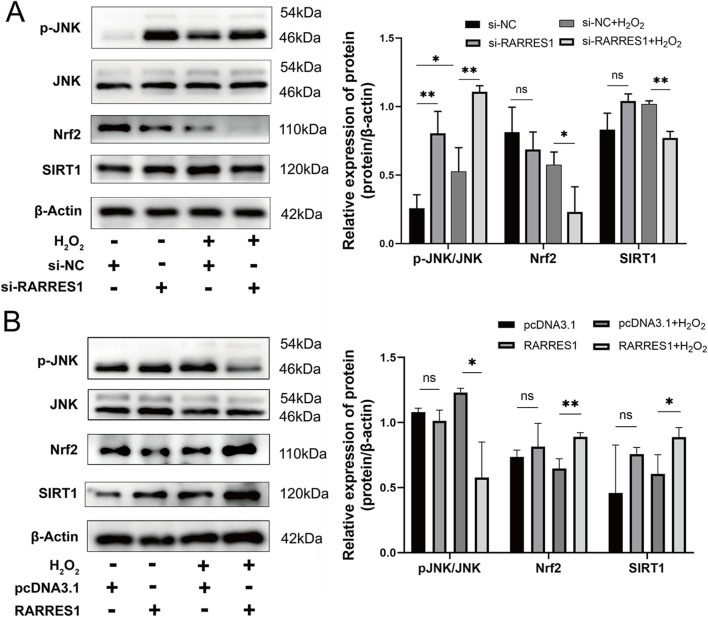
Mechanistic Role of RARRES1 in ARPE-19 Cells Under Oxidative Stress. **(A)** Western blot analysis revealed that knockdown of RARRES1 in ARPE-19 cells under oxidative stress conditions led to activation of phosphorylated JNK (p-JNK) and suppression of Nrf2 and SIRT1 expression. **(B)** In contrast, RARRES1 overexpression reversed these effects, suggesting a protective regulatory mechanism. Protein levels were normalized to β-actin, and mRNA levels to GAPDH. Data are presented as mean ± SD. *P < 0.05, **P < 0.01, ns = not significant.

Western blot analysis was conducted to explore the molecular mechanisms behind RARRES1’s antioxidative action, focusing on the JNK signaling pathway and proteins associated with oxidative stress. The results revealed that si-RARRES1 transfection significantly increased the expression of p-JNK, while notably suppressing the levels of both Nrf2 and SIRT1 in ARPE-19 cells under oxidative stress, compared to the si-NC control group (Figure 5A). In contrast, overexpression of RARRES1 in H_2_O_2_-treated cells significantly inhibited p-JNK activation and enhanced the expression of Nrf2 and SIRT1 relative to the pcDNA3.1 controls (Figure 5B). This suggests that RARRES1 exerts its antioxidative effects by modulating key stress-response pathways, suppressing pro-apoptotic JNK signaling while promoting protective Nrf2 and SIRT1 activity.

### RARRES1 inhibited HUVECs viability, migration, and tube formation *in vitro*

To investigate the effects of RARRES1 on angiogenesis, HUVECs were transfected with siRNA targeting RARRES1 and the pcDNA3.1 RARRES1 plasmid. Immunofluorescence confirmed the expression of RARRES1 in HUVECs (Supplementary Figure S3). Transfection efficiency was evaluated via Western blotting and qPCR. As shown in Supplementary Figure S4, RARRES1 expression was lower in the si-RARRES1 groups compared to the si-NC groups, and higher in the RARRES1 groups compared to the pcDNA3.1 groups, confirming successful transfection.

**FIGURE 6:**
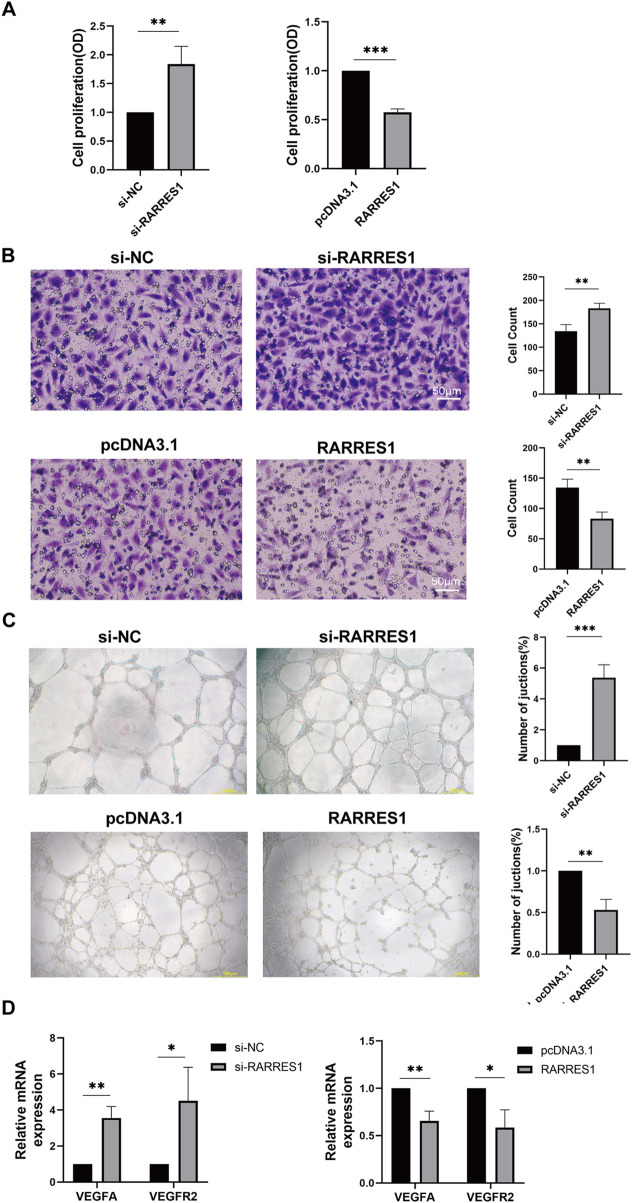
The Role of RARRES1 in HUVECs. **(A)** Cell viability assessed by CCK-8 assay in four experimental groups: RARRES1 knockdown (si-RARRES1), siRNA negative control (si-NC), RARRES1 overexpression (RARRES1), and vector control (pcDNA3.1). RARRES1 knockdown significantly promoted HUVEC viability. **(B)** Cell migration was increased in HUVECs transfected with RARRES1-specific siRNA compared to the si-NC group. Scale bar = 50 μm. **(C)** Tube formation was enhanced in HUVECs following RARRES1 knockdown. Scale bar = 200 μm. **(D)** mRNA levels of pro-angiogenic factors were upregulated in RARRES1 knockdown cells and downregulated in RARRES1-overexpressing cells. mRNA levels were normalized to GAPDH. Data are presented as mean ± SD; n = 3. *P < 0.05, **P < 0.01, ***P < 0.001.

Cell viability was assessed using CCK-8 assays, revealing that knockdown of RARRES1 promoted the viability of HUVECs compared to the si-NC group, while overexpression of RARRES1 inhibited HUVEC viability compared to the pcDNA3.1 group (Figure 6A). The si-RARRES1 group exhibited a markedly greater number of migrating cells compared to the si-NC group, while RARRES1 overexpression markedly suppressed HUVEC migration (Figure 6B). In the tube formation assay, knockdown of RARRES1 increased the number of vessel junctions, whereas overexpression of RARRES1 reduced the number of vessel junctions (Figure 6C). Knockdown of RARRES1 resulted in upregulation of VEGFA and VEGFR2 expression, whereas RARRES1 overexpression downregulated their mRNA levels (Figure 6D). Collectively, these results demonstrate that RARRES1 negatively regulates key processes involved in angiogenesis, highlighting its potential role as an anti-angiogenic factor.

### RARRES1 suppresses the activity and growth of mouse CNV

**FIGURE 7:**
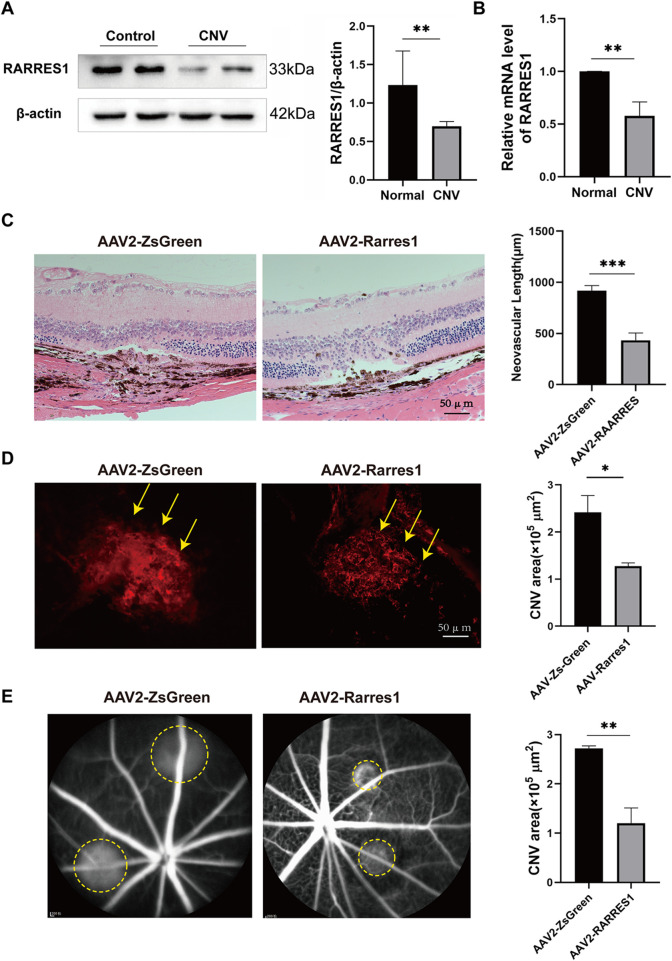
Expression and Functional Role of RARRES1 in a CNV Mouse Model. **(A,B)** Western blot and qPCR analyses revealed significantly decreased RARRES1 protein and mRNA expression levels in the retina–RPE–choroid complex of CNV model mice. Protein and mRNA levels were normalized to β-actin and GAPDH, respectively. **(C)** Hematoxylin and eosin (HE) staining revealed significantly reduced lesion length and area in the AAV2-RARRES1 group compared to the AAV2-ZsGreen control group. **(D)** IB4 immunofluorescence staining demonstrated smaller neovascular lesion areas in the AAV2-RARRES1 group (yellow arrows). **(E)** Representative late-phase fundus fluorescein angiography (FFA) images taken 1 week after laser induction show smaller lesion areas and reduced vascular leakage (indicated by yellow circles) in the AAV2-RARRES1 group compared to controls. Scale bar = 50 μm. Data are presented as mean ± SD; n = 3. *P < 0.05, **P < 0.01, ***P < 0.001.

We first assessed the expression pattern of RARRES1 in the retina-choroid tissues of mice. RARRES1 was predominantly localized to the RPE layer and within CNV lesions, with its expression markedly reduced in the CNV group compared to the control (Supplementary Figure S5). To explore how RARRES1 expression changes *in vivo*, we established a laser-induced CNV mouse model. Western blotting and qPCR analyses revealed a significant downregulation of RARRES1 at both the protein (Figure 7A) and mRNA (Figure 7B) levels in the CNV group compared to normal mice, suggesting that suppression of RARRES1 might be involved in the pathogenesis of CNV.

Next, to investigate the role of RARRES1 in CNV formation, we used adeno-associated virus type 2 (AAV2) to construct a RARRES1 overexpression vector and a negative control. C57BL/6J mice were intravitreally injected with AAV2, and 4 weeks later, Western blotting and qPCR confirmed successful overexpression of RARRES1 in the retina-RPE-choroid tissues, as evidenced by significantly increased protein and mRNA levels compared to the control group (Supplementary Figure S6).

After confirming robust transgene expression, laser-induced CNV was induced. HE staining showed that both the length and area of CNV were substantially reduced in the AAV2-RARRES1 group compared to the control group (Figure 7C) 7 days after the model was established. CNV activity and lesion size were compared between the AAV2-RARRES1 and AAV2-ZsGreen control groups. AAV2-RARRES1 group had a smaller CNV area and volume compared to the AAV2-ZsGreen group, as shown by immunostaining with IB4 (Figure 7D). Reduced vascular leakage was found in the AAV2-RARRES1 group compared to the AAV2-ZsGreen group in the late phase of FFA (Figure 7E). Interestingly, it was found that RARRES1 knockdown reduced Occludin expression in HUVECs, whereas its overexpression increased Occludin expression (Supplementary Figure S7). These *in vivo* findings firmly establish that RARRES1 suppresses CNV progression, validating its potential therapeutic value in nAMD.

## Discussion

This study offered both *in vivo* and *in vitro* evidence demonstrating that RARRES1 (transmembrane isoform) is markedly reduced during the development of nAMD. This was demonstrated in oxidative stress-induced RPE injury models and laser-induced CNV mouse models. *In vitro*, RARRES1 overexpression attenuated H_2_O_2_-induced oxidative damage in ARPE-19 cells, underscoring its cytoprotective role against oxidative stress. Additionally, RARRES1 overexpression inhibited key angiogenic behaviors of vascular endothelial cells, including cell viability, migration, and tube formation. Functional analyses further demonstrated that RARRES1 overexpression effectively suppressed CNV formation *in vivo*. Collectively, these results reveal RARRES1 as a new modulator of nAMD progression through its combined cytoprotective and anti-angiogenic actions, underscoring its promise as a target for nAMD therapy.

RPE is essential for maintaining the structural integrity and function of the outer retina, particularly through its nutritional support to photoreceptors. While the biological roles of RPE cells are well established, the association between RARRES1 and RPE function has not been previously characterized. In this study, we present the first evidence of RARRES1 expression in ARPE-19 cells and demonstrate its significant downregulation under oxidative stress conditions, implicating its potential role in the oxidative stress response. Functionally, RARRES1 overexpression inhibited H_2_O_2_-induced apoptosis and ROS accumulation, reduced the expression of inflammatory and angiogenic factors, and restored tight junction integrity via upregulation of Occludin. These protective effects collectively suggest that RARRES1 may act as a critical regulator of oxidative stress-mediated injury in RPE cells.

Oxidative stress is widely recognized as a major contributing factor in the pathogenesis of AMD, particularly through its impact on RPE cells. RPE cells are metabolically active and play a crucial role in maintaining retinal homeostasis; however, their high oxygen consumption and exposure to light-induced oxidative stress make them particularly vulnerable to damage (Beatty et al., 2000; Jarrett and Boulton, 2012). Increasing evidence suggests that RPE dysfunction caused by oxidative damage represents an initiating event in both dry and neovascular (wet) forms of AMD (Datta et al., 2017). While oxidative stress can also influence endothelial cell behavior, such as promoting angiogenesis, its contribution in this context is considered a secondary response to RPE-derived inflammatory and proangiogenic signaling (Ambati and Fowler, 2012). Therefore, in this study, we selected ARPE-19 cells as a relevant *in vitro* model to investigate the functional role of RARRES1 under oxidative conditions. Our findings support the notion that RARRES1 may serve as a protective factor against oxidative injury in RPE cells and potentially modulate the early pathological changes that precede choroidal neovascularization. These results align with the growing body of research emphasizing the central role of oxidative stress-induced RPE dysfunction in AMD pathophysiology.

Oxidative stress plays a pivotal role in damaging RPE cells, leading to dysfunction and triggering different types of programmed cell death (Cornebise et al., 2023). Of these pathways, apoptosis is broadly acknowledged as the primary mode of RPE cell death in response to oxidative stress (Hanus et al., 2015). Although the apoptotic regulatory role of RARRES1 in RPE cells has not been previously described, it has demonstrated notable but tissue-specific functions in other cell types. For example, RARRES1 has been reported to promote apoptosis in renal podocytes and various tumor cells (Chen et al., 2020; Chen A. et al., 2021; Feng et al., 2024). In contrast, RARRES1 acts as an anti-apoptotic factor in inflammatory breast cancer, where it facilitates cell survival and promotes proliferation and migration (Wang et al., 2013). In this study, we observed that RARRES1 markedly inhibited apoptosis and enhanced the viability of ARPE-19 cells, primarily through the reduction of ROS levels, thereby mitigating oxidative stress-induced cellular damage.

Moreover, oxidative stress is well known to elicit a chronic inflammatory response, which contributes to retinal damage and disease progression (Ahmed et al., 2023; Kaur and Singh, 2021). RARRES1 has been implicated in inflammatory conditions such as pterygium (Suarez et al., 2021), psoriasis, and atopic dermatitis (Seiringer et al., 2024). In our study, RARRES1 overexpression markedly suppressed the expression of pro-inflammatory cytokines in H_2_O_2_-treated cells. In aging eyes, oxidative stress has also been shown to impair RPE barrier integrity, thereby facilitating the development of CNV (Gupta et al., 2023). Our findings revealed that RARRES1 upregulates the expression of the tight junction protein occludin, indicating its potential role in maintaining RPE barrier function under oxidative stress conditions. Collectively, These findings indicate that RARRES1 contributes to protecting RPE cells from oxidative stress-induced inflammation and barrier impairment *in vitro*.

The MAPK family consists of three major members: ERK, JNK, and p38 (Cargnello and Roux, 2011). ERK is predominantly associated with cell proliferation, differentiation, and survival, while JNK and p38 are more closely linked to apoptosis and cellular stress responses (Wada and Penninger, 2004). In our study, we evaluated the expression of these MAPK components in H_2_O_2_-treated cells and observed that RARRES1 overexpression significantly reduced the phosphorylation of JNK (p-JNK), whereas no notable changes were detected in the activation of ERK or p38.

Furthermore, we examined the expression of key antioxidant regulators. SIRT1 is known to protect RPE cells from oxidative injury (Chen W. et al., 2021), mitigate inflammation and apoptosis in H_2_O_2_-stressed ARPE-19 cells (Al Sabaani, 2020), and inhibit choroidal neovascularization (Zhang et al., 2015). Nuclear factor erythroid 2-related factor 2 (Nrf2), a redox-sensitive transcription factor, is a well-established downstream target of SIRT1 that initiates the transcription of antioxidant and anti-inflammatory genes (Li et al., 2021). Nrf2 is essential for protecting RPE cells from oxidative damage and has been identified as a promising therapeutic target for AMD (Bellezza, 2018; Hui et al., 2020).

In the current study, RARRES1 overexpression led to increased expression of both SIRT1 and Nrf2, along with the suppression of p-JNK activation in H_2_O_2_-treated ARPE-19 cells. Our results demonstrate that RARRES1 may protect RPE cells from oxidative stress-induced damage via the JNK/SIRT1/Nrf2 signaling axis, providing insight into its potential as a therapeutic target for AMD.

Angiogenesis is a complex process involving a series of events, such as proliferation and tube formation (Carmeliet and Jain, 2011). However, the relationship between RARRES1 and angiogenesis remains poorly understood. In 2017, Roy et al. (2017) were the first to suggest that RARRES1 plays a role in regulating angiogenesis by demonstrating its inhibitory effect on tube formation in HUVECs in the context of prostate cancer. While this study was a significant step forward, it provided limited mechanistic insight, as it did not address other key angiogenic processes.

In our current study, we not only confirmed Roy et al.'s initial findings but also extended the investigation by examining the role of RARRES1 on both the migration and viability of HUVECs, following its knockdown and overexpression. Additionally, we found that RARRES1 overexpression resulted in the downregulation of key angiogenic factors, including VEGFA and VEGFR2, at the molecular level. These findings collectively demonstrate that RARRES1 effectively suppresses angiogenesis *in vitro*. Moreover, this aligns with our *in vivo* observations, where RARRES1 significantly inhibited CNV formation.

Nonetheless, the exact molecular mechanisms through which RARRES1 regulates ARPE-19 and HUVEC cells are yet to be fully understood. Although this study examined the role of RARRES1 in ARPE-19 cells, HUVECs, and CNV, its wider biological implications in other cell types require further exploration.

## Conclusion

We show that RARRES1 effectively shields RPE cells from oxidative damage by reducing ROS production and inflammation, thus enhancing cell viability. Moreover, RARRES1 inhibits angiogenesis by suppressing tube formation, migration and viability of vascular endothelial cells. Additionally, RARRES1 reduces CNV formation *in vitro*. These findings provide valuable insights into the mechanisms underlying the onset and progression of nAMD, positioning RARRES1 as a promising therapeutic target for CNV.

## Data Availability

The original contributions presented in the study are included in the article/Supplementary Material, further inquiries can be directed to the corresponding author.
