# Supplementary material for: RARRES1 attenuates H2O2-induced RPE cell injury and inhibits choroidal neovascularization
Source: Front Physiol. 2025 Oct 16;16:1641653. doi: 10.3389/fphys.2025.1641653 (PMC12571599; doi:10.3389/fphys.2025.1641653)
Supplement: Supplementary file 1 [file Supplementaryfile1.docx]

**Supplementary materials**

| Table S1. Clinical characteristics of the participants in nAMD and cataract groups. | | | | |
| --- | --- | --- | --- | --- |
| Property | nAMD (n=8) | Cataract (n=8) | *χ^2^/t* | *P value* |
| Age (years) | 67±5.4 | 68±4.5 | 0.50 | 0.623 |
| Sex (male/female) | 3/5 | 4/4 | 0.254 | 0.637 |
| BMI (kg/m^2^) | 24.95±0.90 | 24.88±1.10 | 0.15 | 0.883 |
| Smoking (yes/no) | 25% | 25% | 0 | 1 |
| Alcohol (yes/no) | 12.5% | 25% | 0.41 | 0.522 |

Data were presented as mean ± SD.

| Table S2. PCR primers. | |
| --- | --- |
| Gene name | Primer sequences (5’→ 3’) |
| RARRES1-mouse | F: AGAGCAATACAACCCCGAGC |
|  | R: ATCACGTATGAGCTGCCGAG |
| GAPDH-mouse | F: TGTGTCCGTCGTGGATCTGA |
|  | R: TTGCTGTTGAAGTCGCAGGA |
| RARRES1-human | F: CTAGTGTGAGGCAGTGGAAAACC |
|  | R: GACCAAGTGAATGCGACAGG |
| IL-6-human | F: GTCAACTCCATCTGCCCTTCAG |
|  | R: GGTCTGTTGTGGGTGGTATCCT |
| IL-8-human | F: TCTCTTGGCAGCCTTCCTGA |
|  | R: CGCAGTGTGGTCCACTCTCA |
| IL-1β-human | F: ACAGATGAAGTGCTCCTTCCA |
|  | R: GTCGGAGATTCGTAGCTGGAT |
| MCP1-human | F: GTCTCTGCCGCCCTTCTGT |
|  | R: TTGCATCTGGCTGAGCGAG |
| TNFɑ-human | F: GTGACAAGCCTGTAGCCCATGTTA |
|  | R: TTATCTCTCAGCTCCACGCCATTG |
| VEGFA-human | F: CTCACACACACACCAACCAGG |
|  | R: GAAGAAGCAGCCCATGACAG |
| VEGFR2-human | F: CTCACACACACACCAACCAGG |
|  | R: GAAGAAGCAGCCCATGACAG |
| GAPDH-human | F: TGCACCACCAACTGCTTAGC |
|  | R: GGCATGGACTGTGGTCTGAG |


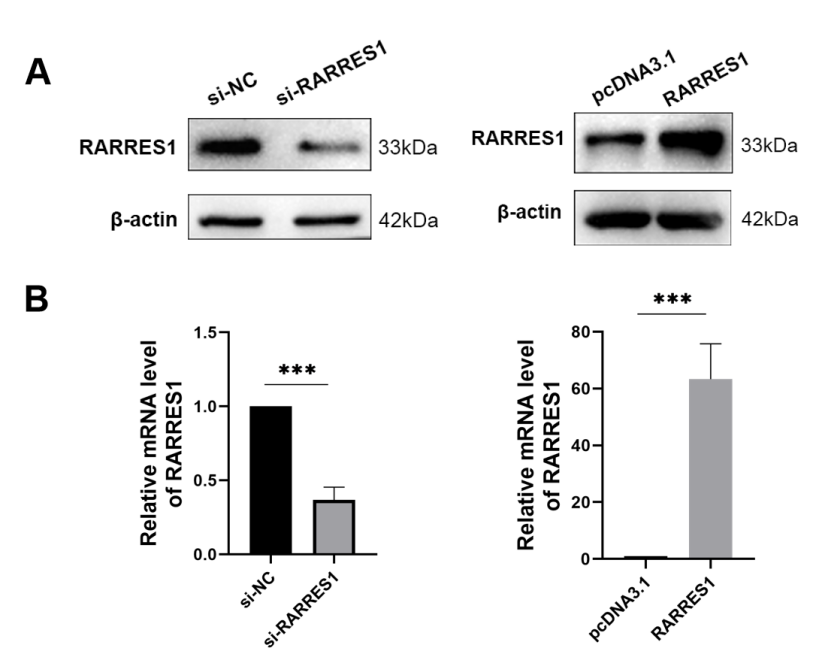


Figure S1. Expression of RARRES1 in ARPE-19 cells after siRNA and pcDNA3.1 transfection. (A-B) Western blot and qRT-PCR detection of knockdown and overexpression efficiency of siRNA and pcDNA3.1 in ARPE-19 cells. Protein levels are normalized against β-actin. The mRNA levels are normalized by GAPDH. Data were displayed with mean±SD. ***P < 0.001.


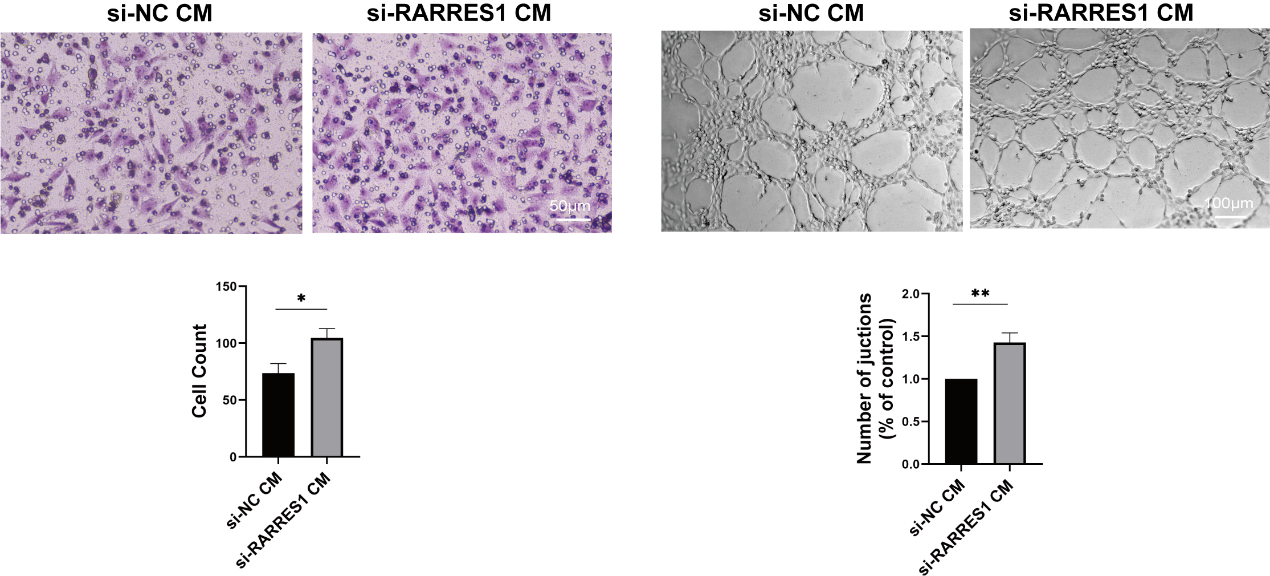


Figure S2. The effects of the conditioned medium from RARRES1 knockdown cells on the angiogenic potential of HUVECs. Data were displayed with mean±SD. *P<0.05, **P<0.01.


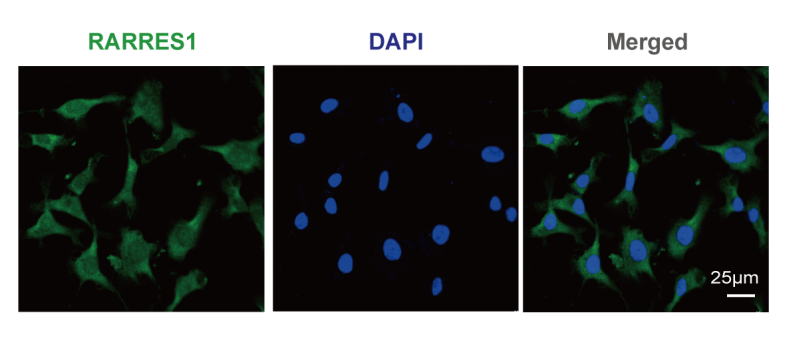


Figure S3. Immunofluorescence images showing signal intensity of RARRES1 in HUVECs.


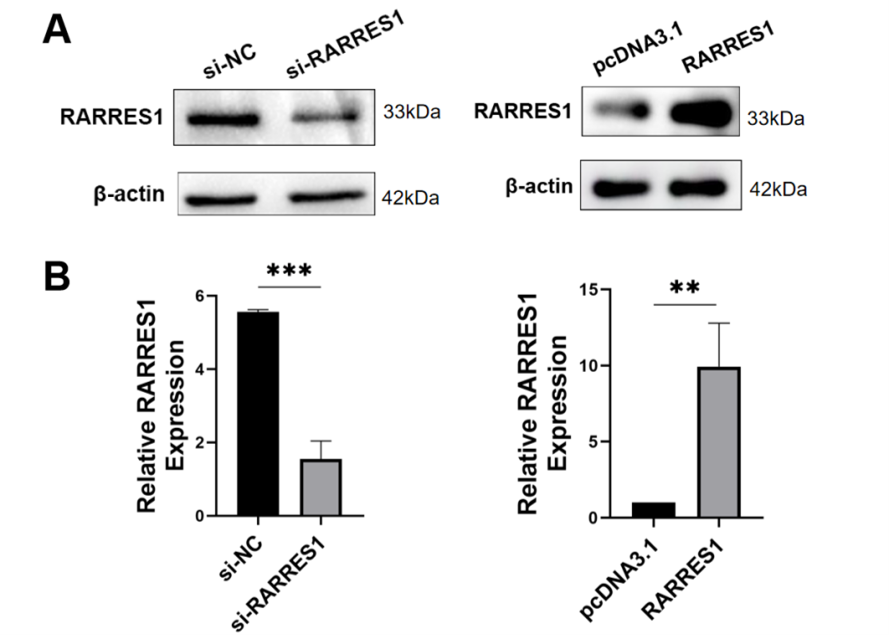


Figure S4. Expression of RARRES1 in HUVECs after siRNA and pcDNA3.1 transfection. (A-B) Western blot and qRT-PCR detection of knockdown and overexpression efficiency of siRNA and pcDNA3.1 in HUVECs. Protein levels are normalized against β-actin. The mRNA levels are normalized by GAPDH. Data were displayed with mean±SD. **P < 0.01, ***P < 0.01.


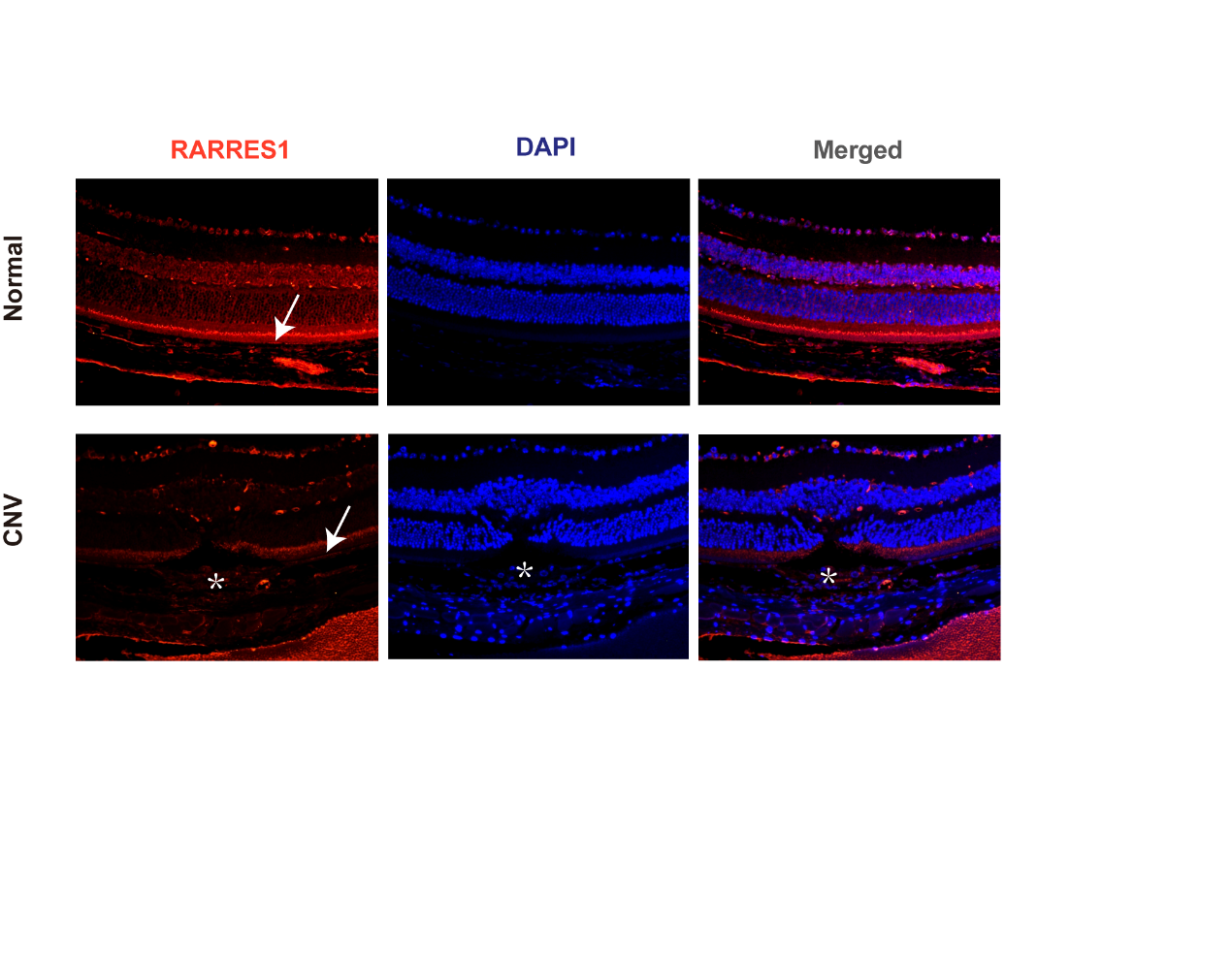


Figure S5. Immunofluorescence staining of paraffin-embedded mouse retinal sections demonstrated RARRES1 localization predominantly in the retinal pigment epithelium (RPE) layer (arrowheads) and within the choroidal neovascularization (CNV) lesions (asterisk). A marked reduction in fluorescence intensity was observed in the CNV group compared to the control.


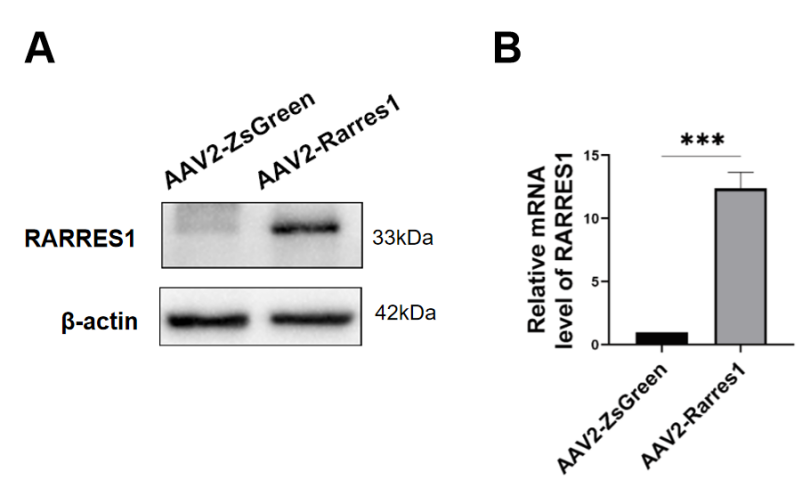


Figure S6. Overexpression of RARRES1 in ARPE-19 cells detected by Western blot and qRT-PCR. The protein expression level of RARRES1 was significantly upregulated in the AAV2-Rarres1 group compared with the AAV2-ZsGreen control group. (B) Consistently, the mRNA expression level of RARRES1 was also markedly elevated in the AAV2-Rarres1 group. Data were displayed with mean±SD. ***P<0.001.


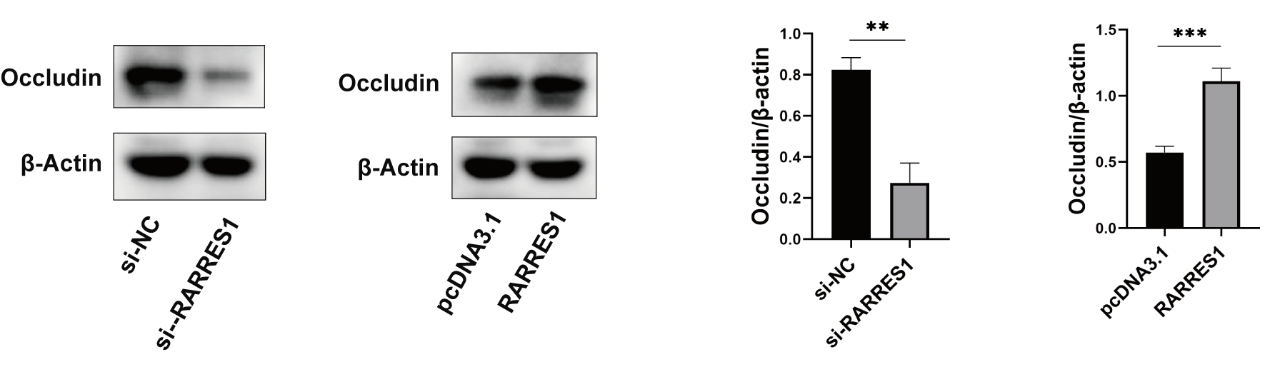


Figure S7. The effect of RARRES1 gene knockdown or overexpression on Occludin protein expression in HUVECs. Data were displayed with mean±SD. **P<0.01, ***P<0.001.
